# Urinary miRNA Expression in Pre-Eclampsia During Early and Mid-Pregnancy

**DOI:** 10.3390/ncrna10060061

**Published:** 2024-12-02

**Authors:** Roman A. Illarionov, Anastasia R. Maltseva, Olga V. Pachuliia, Tatiana B. Postnikova, Elena S. Vashukova, Anastasiia K. Popova, Yulia A. Nasykhova, Olesya N. Bespalova, Andrey S. Glotov

**Affiliations:** 1 Department of Genomic Medicine, D.O. Ott Research Institute for Obstetrics, Gynecology, and Reproduction, St. Petersburg 199034, Russia; 2 Department of Genetics and Biotechnology, St. Petersburg State University, St. Petersburg 199034, Russia

**Keywords:** miRNA, pregnancy, urine, pre-eclampsia, high-throughput sequencing

## Abstract

**Background:** Pre-eclampsia (PE) is a serious condition affecting 2–8% of pregnancies worldwide, leading to high maternal and fetal morbidity and mortality. MicroRNAs (miRNAs), small non-coding RNA molecules, have emerged as potential biomarkers for various pregnancy-related pathologies, including PE. MiRNAs in plasma and serum have been extensively studied, but urinary miRNAs remain underexplored, especially during early pregnancy. This study aimed to investigate the urinary miRNA expression profiles in women with pre-eclampsia during the first and second trimesters. **Materials and Methods:** A prospective study was conducted using 48 urine samples from 24 pregnant women (n = 12 pre-eclampsia and n = 12 controls). Urine samples were collected in the first (9–13 weeks) and second (22–24 weeks) trimesters. MiRNA isolation, library preparation, and high-throughput sequencing were performed, followed by differential expression and enrichment analyses. **Results:** In the first trimester, five miRNAs were dysregulated in PE in comparison with the control group (hsa-miR-184, hsa-miR-203a-3p, hsa-miR-205-5p, hsa-miR-223-3p—downregulated; hsa-miR-1-3p—upregulated). In the second trimester, hsa-miR-205-5p and hsa-miR-223-3p were downregulated, and hsa-miR-9-5p, hsa-miR-1-3p, and hsa-miR-206 were upregulated. **Conclusions:** Our study identified differentially expressed miRNAs in the urine of pre-eclamptic patients during early pregnancy. These findings suggest that specific urinary miRNAs could serve as non-invasive biomarkers for the early detection and risk assessment of pre-eclampsia. The changes in the level of differential expression of miRNAs during gestation highlight their role in the progression of PE. Further research and validation with a larger cohort are needed to explore their clinical potential for improving maternal and fetal outcomes through early intervention.

## 1. Introduction

Pre-eclampsia (PE) is a severe pathological condition that occurs after the 20th week of pregnancy and disappears a few days after delivery. PE is followed by a triad of major symptoms: arterial hypertension, proteinuria, and edema. According to the World Health Organization (WHO), PE affects 2–8 percent of all pregnancies worldwide and is characterized by a high incidence of maternal and child morbidity and mortality [1]. Early diagnosis and prompt treatment are essential to prevent both maternal and neonatal complications.

MicroRNAs (miRNAs) are considered to be biomarkers of interest in many pathologic conditions in pregnancy, including PE [2]. Researchers pay the most attention to the study of miRNAs after the onset of complications, which is important for understanding the pathogenesis and regulatory mechanisms of PE. However, this approach limits the potential for early detection and prevention. It has been shown that PE begins in the first trimester of pregnancy when disruptions occur in the interaction between fetal (due to inadequate trophoblast invasion) and maternal vessels (due to the improper remodeling of spiral arteries). This results in arterial spasms, a reduction in blood flow, and the initiation of a complex cascade of pathological responses, ultimately manifesting in PE [3].

Studies have suggested that circulating microRNAs in early pregnancy could serve as potential biomarkers for pre-eclampsia. However, most studies have focused on miRNAs in plasma and serum [4,5,6]. Circulating miRNAs have also been detected in other biological fluids, such as saliva, gastric juice, and urine [7].

Urine is one of the most accessible and non-invasive biological fluids; it can be collected by the patient in relatively large volumes. Studies have shown that miRNAs in urine are quite stable [8] and can serve as potential biomarkers for various cancers [9,10]. Although there are limited studies on urinary miRNAs in pregnancy, Inno and Laan identified placenta-specific miRNAs in urine, indicating the potential of miRNAs as biomarkers for pregnancy complications [11]. Previously, Herrera-Van Oostdam et al. studied the urinary miRNA expression profile in gestational diabetes mellitus throughout gestation [12]. Gan et al. compared the expression of miR-210, miR-155, miR-125b-5p, and miR-125a-5p in urine samples collected before delivery from pre-eclamptic and normal pregnant women, but found no significant differences [13]. However, to our knowledge, no studies have examined urinary miRNAs in PE during early or mid-pregnancy.

We conducted a prospective study of the urinary miRNA expression profile in PE in the first and second trimesters of pregnancy.

## 2. Materials and Methods

### 2.1. Study Participants

Urine samples for this study were sourced from the Biobank “Genofond” at the D.O. Ott Research Institute of Obstetrics, Gynecology, and Reproductology. A total of 48 urine samples were collected from 24 women during two specific gestational periods: the first trimester (9–13 weeks) and the second trimester (22–24 weeks).

The study group (PE) consisted of pregnant women diagnosed with late-onset pre-eclampsia (n = 12). All pregnant women had a symptom complex: arterial hypertension, proteinuria, and edema.

The control group comprised women with uncomplicated, physiological pregnancies. Control samples were collected at gestational ages matched to those of the pre-eclampsia group.

All participants met the following inclusion criteria: age between 25 and 35 years, a singleton pregnancy, and provision of informed consent.

The exclusion criteria for all participants were as follows: extragenital pathology associated with the risk of PE, including obesity, pregestational diabetes mellitus, chronic arterial hypertension, and chronic kidney disease; gestation complications (early toxicosis in pregnant women, a high risk of fetus chromosomal abnormalities, congenital pathologies of fetal development according to the combined first screening); the absence of late pregnancy complications (hepatosis, placental abruption, cervical insufficiency, polyhydramnios, oligohydramnios, and others); and bloodborne infections (hepatitis, HIV, APS and others).

Ethical approval for this study was obtained from the Institutional Review Board of the D.O. Ott Research Institute of Obstetrics, Gynecology, and Reproductology, under protocol No. 97, dated 27 June 2019. All participants provided written informed consent prior to enrollment, which included consent for the use of their personal and medical data. The study was conducted in compliance with the principles outlined in the Declaration of Helsinki.

### 2.2. Sample Preparation

Urine samples were collected in sterile urine containers and kept at 4 °C for no longer than 2 h. A volume of 40 mL from each sample was transferred into 50 mL tubes and centrifuged at 3000× *g* for 20 min at 4 °C. The resulting supernatant was carefully decanted into a sterile tube and thoroughly mixed. Immediately after centrifugation, the urine samples were aliquoted into 4 mL cryotubes, then promptly frozen and stored at −80 °C until further analysis.

### 2.3. MiRNA Isolation and Library Preparation for Sequencing

miRNA was isolated from 200 µL of urine using the miRNeasy Serum/Plasma Advanced Kit (Qiagen, Hilden, Germany), following the manufacturer’s instructions. The extracted RNA was resuspended in 12 µL of RNase-free water and stored at −80 °C until further use in library preparation.

miRNA libraries were constructed using the QIAseq miRNA Library Kit (Qiagen, Hilden, Germany) according to the manufacturer’s protocol. In brief, RNA samples were ligated with 3′ and 5′ adapters, followed by reverse transcription to generate cDNA. The cDNA was purified using magnetic beads and eluted with 17 µL of nuclease-free water. These cDNA samples served as templates for subsequent PCR amplification. Unique barcodes were added to each sample during PCR to facilitate the pooling of libraries for sequencing. The resulting PCR products were cleaned with magnetic beads and eluted in 25 µL of nuclease-free water. The yield, size distribution, and molar concentration of the DNA libraries were assessed using a 2200 TapeStation Instrument with High Sensitivity D1K ScreenTape and Reagents (Agilent Technologies, Santa Clara, CA, USA). The required quantity of libraries for sequencing was determined using a Qubit 2.0 Fluorometer with a dsDNA High-Sensitivity Kit (Thermo Fisher Scientific, Waltham, MA, USA), following the manufacturer’s guidelines.

### 2.4. Illumina Sequencing

All prepared libraries were sequenced together using a HiSeq 2500 platform (Illumina, San Diego, CA, USA) with single-end 75 bp reads, adhering to the manufacturer’s protocol.

### 2.5. Clinical Data Analysis

Clinical data were analyzed using Statistica 10.0 software (StatSoft, Inc., Tulsa, OK, USA). Continuous variables are expressed as means ± standard error, while categorical variables are presented as counts (percentages).

### 2.6. Sequencing Data Analysis

Small-RNA sequencing data were processed using the GeneGlobe Data Analysis Center (https://geneglobe.qiagen.com/us/analyze, accessed on 12 September 2024). This included adapter trimming and mapping to miRBase to generate raw count data. The average high-throughput sequencing read counts for each RNA category were calculated for the urine samples from pregnant women, with 12 samples per group. Differential expression analysis was performed on the raw count matrix from 24 individual samples using the DESeq2 R package [14]. The median-of-ratios normalization method, as implemented in DESeq2, was applied. miRNAs were considered differentially expressed if the adjusted *p*-value was <0.05 and the absolute value of log2(fold change) exceeded 1.5. Target prediction for miRNAs was conducted using the TargetScan v8.0 database (https://www.targetscan.org/vert_80/, accessed on 12 September 2024) [15]. Gene enrichment analysis was performed using the DIANA-miRPath v4.0 tool [16].

## 3. Results

### 3.1. Characteristics of Objectives

Using the inclusion criteria, 24 pregnant women were selected: 12 in the control group (CTRL), and 12 in the group with pre-eclampsia (PE). The characteristics of the participants and their pregnancy outcomes are presented in Table 1.

The groups were comparable in terms of age and body mass index (BMI) (*p* > 0.05). At the same time, statistically significant differences were observed in blood pressure and proteinuria, as well as the presence of edema, and Apgar scores between the group of pregnant women with PE and the control group (*p* < 0.05). Statistically significant differences were also found in the method and gestational ages of delivery and the weight and height of newborns in pregnant women with PE compared to the group without pregnancy complications (*p* < 0.05).

### 3.2. Comparison of miRNA Profiles in Urine Between Pre-Eclampsia and Control Groups

**Figure 1:**
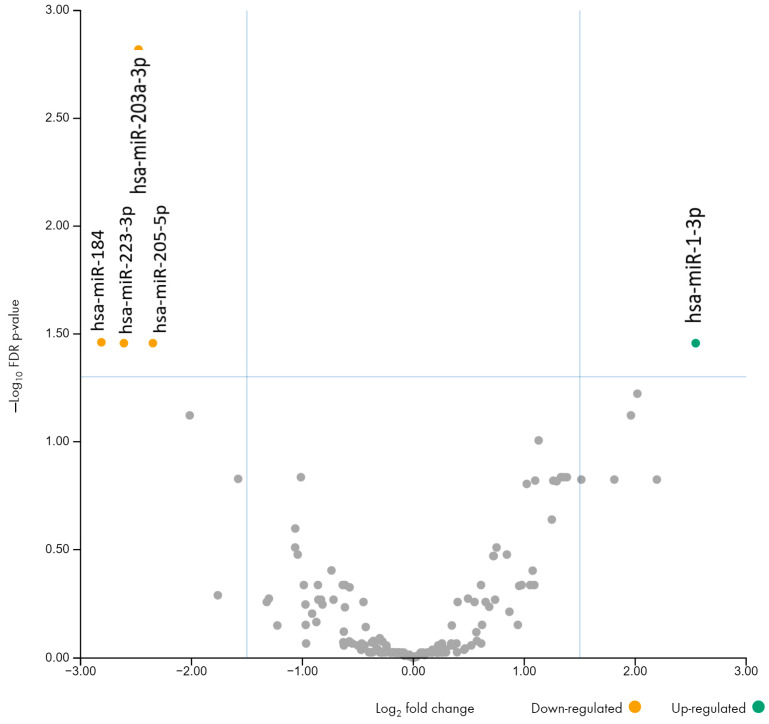
Volcano plot (first trimester).

In total, 431 miRNAs were detected in the first trimester and 499 in the second trimester. Among all miRNAs identified in the first trimester, five were differentially expressed: there were four microRNAs with decreased expression (hsa-miR-184, hsa-miR-203a-3p, hsa-miR-205-5p, hsa-miR-223-3p) and one microRNA with increased expression (hsa-miR-1-3p) in the urine samples of patients who later developed PE (FDR < 0.05, |log2FC| > 1.5) (Table 2, Figure 1).

**Figure 2:**
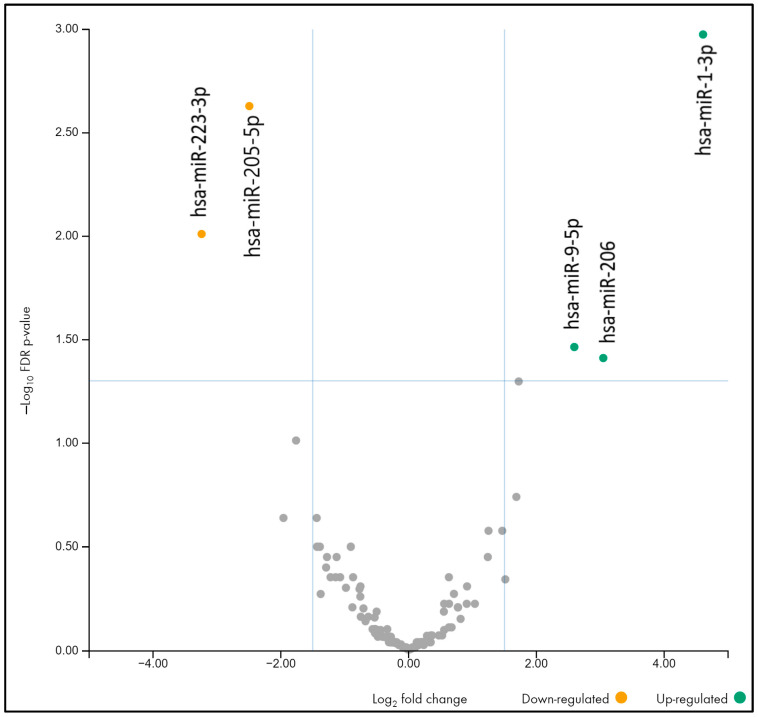
Volcano plot (second trimester).

In the second trimester, two miRNAs were downregulated (hsa-miR-205-5p, hsa-miR-223-3p) and three were upregulated (hsa-miR-9-5p, hsa-miR-1-3p, hsa-miR-206) (FDR < 0.05, |log2FC| > 1.5) (Table 3, Figure 2).

Interestingly, for three microRNAs, hsa-miR-1-3p (upregulated), hsa-miR-223-3p, and hsa-miR-205-5p (downregulated), differences are observed in both the first and second trimesters, with the difference becoming more pronounced in the second trimester, which allows us to consider changes in the level of these three microRNAs in the urine of pregnant women as new possible early biomarkers of PE.

### 3.3. Target Prediction and Gene Ontology Analyses

Gene enrichment analysis of the predicted target genes revealed 107 KEGG pathways that were significantly overrepresented among potential miRNA targets in the first and second trimesters. Table 4 and Table 5 present the top 15 enriched KEGG pathways for each trimester. The KEGG enrichment analysis indicated that most of the differentially expressed miRNAs targeted genes involved in several key signaling pathways, including the MAPK, Ras, Rap1, and Hippo signaling pathways, and EGFR tyrosine kinase inhibitor resistance (Table 4 and Table 5).

## 4. Discussion

The search for early biomarkers is essential for predicting the onset of pre-eclampsia and improving clinical outcomes through early intervention. In recent years, significant progress has been made in identifying early biomarkers for predicting pre-eclampsia [6].

The early detection of pre-eclampsia has historically focused on clinical and biochemical markers. These include uterine artery Doppler ultrasound, which can indicate poor placentation, and biochemical markers such as soluble Fms-like tyrosine kinase-1 (sFlt-1), placental growth factor (PlGF), and soluble endoglin (sEng). These markers play key roles in angiogenesis and are dysregulated in pre-eclampsia. The sFlt-1/PlGF ratio is the most validated biochemical marker for pre-eclampsia diagnosis and prediction, with a high sFlt-1/low PlGF profile associated with the disease’s onset. Decreased levels of Pregnancy-associated plasma protein-A (PAPP-A) in the first trimester may indicate a higher risk of pre-eclampsia [17].

However, these biomarkers, while valuable, often fail to detect the condition early enough or with sufficient sensitivity. This has spurred research into novel biomarkers, especially at the molecular level, including circulating miRNAs.

In our research, we identified the urinary miRNA profile in pre-eclampsia in the first and second trimesters through high-throughput sequencing. Among 431 and 499 urinary miRNAs in the first and second trimesters of pregnancy, respectively, we found 6 miRNAs from C19MC and 5 miRNAs from C14MC, both of which are placenta-specific microRNA clusters playing a significant role in the development of PE [18]. Previously, Inno and Laan found eight transcripts from C19MC and one transcript from C14MC obtained from urine samples of pregnant women [11]. These findings align with our data, confirming active filtration in the maternal–fetal urinary system. In our recent work, we identified 1499 and 1556 plasma miRNAs during the first and second trimesters, respectively, in women at high risk of preterm birth; among the differentially expressed miRNAs, 12 were from C14MC, but none from C19MC. Based on this, plasma is a more diverse source for miRNA research, but urine may also be a potential source for finding early miRNA-based biomarkers in various pregnancy complications [19].

In the first trimester, five miRNAs were dysregulated in PE compared to the control group (hsa-miR-184, hsa-miR-203a-3p, hsa-miR-205-5p, hsa-miR-223-3p—downregulated; hsa-miR-1-3p—upregulated). In the second trimester, hsa-miR-205-5p and hsa-miR-223-3p were downregulated, and hsa-miR-9-5p, hsa-miR-1-3p, and hsa-miR-206 were upregulated. Among all the differentially expressed microRNAs in the two trimesters, three were detected in both (hsa-miR-1-3p (upregulated); hsa-miR-223-3p and hsa-miR-205-5p (downregulated)). Moreover, the differences in the expression of these microRNAs increased with the gestation period (the fold change increased in hsa-miR-1-3p by 317%, in hsa-miR-223-3p by 55%, and in hsa-miR-205-5p by 11%), suggesting that they may have a potentially significant contribution to the development of PE. In addition, we previously found differential expression of miR-1 and miR-223 in the placenta during PE [20], which confirms their influence in the pathogenesis of PE.

The role of specific miRNAs in PE, including hsa-miR-1-3p, hsa-miR-223-3p, and hsa-miR-205-5p, has been increasingly investigated, as their altered expression patterns may serve as early indicators of disease onset or progression. hsa-miR-1-3p is predominantly known for its involvement in cardiovascular and muscle-related functions, particularly in regulating cardiac hypertrophy and muscle differentiation [21]. In the context of pre-eclampsia, hsa-miR-1-3p has been observed to be dysregulated in maternal blood and placental tissue [20,22,23]. Pre-eclampsia is often associated with endothelial dysfunction, and hsa-miR-1-3p plays a role in vascular smooth muscle cell regulation [24]. The dysregulation of hsa-miR-1-3p could contribute to abnormal blood vessel formation and function in the placenta, leading to the hypoxic environment characteristic of PE [25].

PE is often considered an inflammatory disease, with increased pro-inflammatory cytokine levels and an imbalance between the immune system’s regulatory and effector arms [26]. hsa-miR-223-3p has been shown to modulate the inflammatory cascade by targeting key molecules such as NF-κB and interleukin-6 (IL-6). The dysregulation of this miRNA in PE may amplify the inflammatory responses that lead to endothelial dysfunction and maternal hypertension [27]. hsa-miR-223-3p also plays a role in the regulation of immune cell differentiation and function, particularly in monocytes and neutrophils. Altered levels of this miRNA could contribute to the immune maladaptation observed in pre-eclampsia, where an imbalance between pro-inflammatory and regulatory immune cells is thought to contribute to abnormal placental development [28].

hsa-miR-205-5p is involved in the epithelial-to-mesenchymal transition (EMT) and is crucial in maintaining the function of trophoblasts, the specialized cells responsible for placenta formation and maternal–fetal exchange. The proper invasion of trophoblast cells into the maternal decidua is essential for establishing adequate blood flow to the placenta [29]. In pre-eclampsia, trophoblast invasion is often shallow, leading to insufficient placentation. hsa-miR-205-5p has been implicated in the regulation of trophoblast invasion and migration by targeting proteins such as E-cadherin, a molecule critical for cell–cell adhesion [30]. The dysregulation of hsa-miR-205-5p may impair this process, contributing to the poor placentation seen in PE. hsa-miR-205-5p also influences angiogenic signaling pathways, crucial for the development of the placental vasculature. Reduced expression of this miRNA has been associated with impaired vascularization in the placenta, a hallmark of pre-eclampsia that leads to reduced oxygen and nutrient exchange between mother and fetus [31].

hsa-miR-1-3p, hsa-miR-223-3p, and hsa-miR-205-5p are emerging as critical players in the complex pathophysiology of pre-eclampsia. By influencing vascular function, immune responses, and placental development, these miRNAs offer novel insights into the molecular mechanisms underlying this life-threatening condition. Their potential as biomarkers and therapeutic targets holds promise for improving the management of pre-eclampsia, ultimately reducing the burden of this disease on maternal and fetal health. Ongoing research into the specific roles of these miRNAs in pre-eclampsia will further refine our understanding and lead to more effective diagnostic and therapeutic strategies.

We also analyzed the enrichment of differentially regulated miRNA target genes. The enrichment analysis of possible targets of miRNAs showed the effect of differentially expressed miRNAs on the Ras, Rap1, Hippo, MAPK, and EGFR tyrosine kinase inhibitor resistance signaling pathways in both trimesters.

The Hippo signaling pathway is a critical regulator of organ size, tissue homeostasis, and cell proliferation, and has recently been implicated in the pathogenesis of pre-eclampsia [32]. Dysregulation of the Hippo pathway in trophoblast cells plays a central role in placental development and can contribute to abnormal placental formation, one of the hallmarks of pre-eclampsia [33]. Studies suggest that reduced Hippo pathway activity may promote trophoblast invasion and enhance placental development, processes that are often impaired in pre-eclampsia. In normal pregnancy, balanced Hippo signaling ensures proper cytotrophoblast differentiation and the formation of syncytiotrophoblasts, which are essential for nutrient exchange between mother and fetus [34]. Understanding the specific role of Hippo signaling in trophoblast function and placental development offers potential therapeutic avenues for managing pre-eclampsia, as targeting this pathway, including miRNA, could restore normal placental function and mitigate disease progression.

EGFR signaling is important for placental development, especially in trophoblast invasion and endothelial function. Aberrations in this pathway could lead to poor placental perfusion, a key feature of pre-eclampsia. Dysregulated EGFR signaling could result in increased oxidative stress, inflammation, and vascular dysfunction, possibly mediated through Ras and MAPK signaling in PE [35].

The Ras family of GTPases, including Ras and Rap1, regulates key cellular processes such as proliferation, migration, and survival. In pre-eclampsia, abnormal Ras activation is linked to impaired trophoblast invasion, vascular dysfunction, and hypertension, driven by oxidative stress and inflammation. Rap1, essential for endothelial barrier function and angiogenesis, may exhibit reduced activity in pre-eclampsia, leading to defective placental blood flow and endothelial dysfunction, worsening the condition’s symptoms [36].

The MAPK (Mitogen-Activated Protein Kinase) pathway, downstream of Ras, is essential for transmitting signals from the cell surface to the nucleus, where it regulates gene expression and cellular responses to external stimuli. In pre-eclampsia, overactivation of the MAPK pathway has been implicated in the heightened inflammatory response and oxidative stress seen in the disorder [37]. This pathway also plays a significant role in the regulation of vascular tone and smooth muscle contraction, both of which are disrupted in pre-eclampsia, leading to increased blood pressure. Moreover, MAPK signaling is involved in the production of pro-inflammatory cytokines and vasoconstrictors such as endothelin-1, both of which contribute to the pathophysiology of pre-eclampsia [38].

The interaction between EGFR signaling and its resistance mechanisms, along with dysregulation of the Hippo, Ras, Rap1, and MAPK pathways, provides valuable insights into the molecular underpinnings of PE. These signaling pathways not only affect placental development and vascular function, but also represent potential therapeutic targets for mitigating the disease’s progression.

The main limitation of our study is the lack of RT-PCR validation of the miRNA sequencing results. This limitation is primarily due to the lack of a sufficient number of patients to perform validation, and our objective was to generate an exploratory miRNA profile rather than validate individual miRNAs. Although high-throughput sequencing provides robust quantitative data, we recognize that further validation would enhance the reliability of the specific miRNA candidates identified here. Future studies with additional patient numbers and RT-PCR validation are needed to confirm these results and improve reproducibility.

Our study identified differentially expressed miRNAs and associated signaling pathways in pre-eclampsia, providing further insight into the molecular mechanisms driving its pathogenesis. Notably, our findings are consistent with those of previous studies, reinforcing the idea that dysregulated miRNAs and their involvement in key signaling pathways play a crucial role in the development and progression of pre-eclampsia. This alignment with prior research strengthens the validity of our results. Understanding the interactions between these miRNAs and their target pathways offers valuable opportunities for identifying novel biomarkers for early diagnosis and potential therapeutic targets.

## 5. Conclusions

Our study successfully identified differentially expressed miRNAs in the urine of pre-eclamptic patients during the first and second trimesters of pregnancy. These findings offer valuable insights into the early molecular changes associated with pre-eclampsia, suggesting that specific urinary miRNAs could serve as non-invasive biomarkers for early detection and risk assessment of the disease. The differential expression patterns observed across trimesters emphasize the dynamic regulation of miRNAs in the progression of pre-eclampsia. Future research focusing on the functional roles of these miRNAs, along with validation in larger cohorts, will be essential for evaluating their clinical potential in improving maternal and fetal outcomes through early intervention strategies.

## Figures and Tables

**Table 1:** Characteristics summary of the pregnant women included in this study.

Characteristic	CTRL (n = 12)	PE (n = 12)
Age, years	30.67 ± 1.62	32.58 ± 1.38
BMI, kg/m^2^	22.60 ± 0.64	25.2 ± 1.19
Symptoms during the manifestation of PE		
Systolic blood pressure (SBP), mmHg	122.0 ± 7.89	**152.7 ± 4.88**
Diastolic blood pressure (DBP), mmHg	70.9 ± 7.01	**96.82 ± 2.72**
Proteinuria	0 (0.0%)	**12 (100%)**
Edema	2 (16.7%)	**12 (100%)**
Outcomes of current pregnancy		
Method of delivery (Cesarean section)	3 (25.0%)	**6 (50.0%)**
Gestational ages of delivery, weeks	40.00 ± 0.21	**36.76 ± 1.51**
Fetal weight, g	3518.0 ± 116.4	**2916.0 ± 262.4**
Fetal height, cm	51.92 ± 0.40	**48.18 ± 1.51**
Apgar score, 1 min	8.17 ± 0.11	**7.36 ± 0.31**
Apgar score, 5 min	8.58 ± 0.19	**7.82 ± 0.18**

Continuous variables are presented as means ± standard error. Statistically significant differences between the comparison group and the group with the corresponding pathology are highlighted in bold (*p* < 0.05).

**Table 2:** Urinary miRNAs with different levels in the first trimester of the pre-eclampsia group compared to the controls.

miRNA	Fold Change	Adjusted *p*-Value (FDR)
hsa-miR-184	−7.02	0.035
hsa-miR-223-3p	−6.10	0.035
hsa-miR-203a-3p	−5.57	0.001
hsa-miR-205-5p	−5.09	0.034
hsa-miR-1-3p	5.84	0.035

**Table 3:** Urinary miRNAs with different levels in the second trimester of the pre-eclampsia group compared to the controls.

miRNA	Fold Change	Adjusted *p*-Value (FDR)
hsa-miR-205-5p	−5.63	0.002
hsa-miR-223-3p	−9.45	0.010
hsa-miR-9-5p	6.03	0.034
hsa-miR-206	8.27	0.039
hsa-miR-1-3p	24.40	0.001

**Table 4:** The top 15 enriched KEGG pathways across predicted targets of miRNAs with different levels in the first trimester.

Term Name	Term Genes	Target Genes (n)	miRNAs (n)	Adjusted *p*-Value (FDR)
Axon guidance	186	93	4	1.6639 × 10^−10^
MAPK signaling pathway	329	144	4	1.6639 × 10^−10^
Ras signaling pathway	241	112	4	2.6679 × 10^−10^
Rap1 signaling pathway	214	102	4	2.9114 × 10^−10^
Hippo signaling pathway	164	82	5	1.4889 × 10^−9^
Pathways in cancer	555	210	4	6.0789 × 10^−9^
Regulation of actin cytoskeleton	224	100	4	2.9138 × 10^−8^
Focal adhesion	213	93	4	4.0345 × 10^−7^
Proteoglycans in cancer	220	95	4	4.8956 × 10^−7^
Calcium signaling pathway	246	103	4	7.762 × 10^−7^
Bacterial invasion of epithelial cells	80	43	4	2.8427 × 10^−6^
AGE-RAGE signaling pathway in diabetic complications	115	56	4	3.0074 × 10^−6^
EGFR tyrosine kinase inhibitor resistance	82	43	4	6.0919 × 10^−6^
Neurotrophin signaling pathway	124	58	4	8.8447 × 10^−6^
Pancreatic cancer	78	41	4	9.2329 × 10^−6^

**Table 5:** The top 15 enriched KEGG pathways across the predicted targets of miRNAs with different levels in the second trimester.

Term Name	Term Genes	Target Genes (n)	miRNAs (n)	Adjusted *p*-Value (FDR)
Rap1 signaling pathway	214	71	5	4.56713 × 10^−11^
Ras signaling pathway	241	72	5	4.64624 × 10^−9^
Signaling pathways regulating pluripotency of stem cells	156	52	5	2.47369 × 10^−8^
Pathways in cancer	555	128	5	3.48636 × 10^−8^
Regulation of actin cytoskeleton	224	65	5	6.71583 × 10^−8^
MAPK signaling pathway	329	85	5	9.23522 × 10^−8^
EGFR tyrosine kinase inhibitor resistance	82	33	5	1.01508 × 10^−7^
Axon guidance	186	56	5	1.25819 × 10^−7^
Adherens junction	79	32	5	1.25819 × 10^−7^
Hepatocellular carcinoma	177	54	5	1.28565 × 10^−7^
Bacterial invasion of epithelial cells	80	32	5	1.35575 × 10^−7^
Breast cancer	163	50	5	3.32511 × 10^−7^
Hippo signaling pathway	164	50	5	3.8429 × 10^−7^
PI3K-Akt signaling pathway	372	90	5	4.3087 × 10^−7^
Gastric cancer	162	49	5	6.16709 × 10^−7^

## Data Availability

The raw data supporting the conclusions of this article will be made available by the authors on request.
